# Supplementary material for: Content-rich biological network constructed by mining PubMed abstracts
Source: BMC Bioinformatics. 2004 Oct 8;5:147. doi: 10.1186/1471-2105-5-147 (PMC528731; doi:10.1186/1471-2105-5-147)
Supplement: Additional File 2 — The original results of the above study (non-essential files are deleted to keep the file size under the limit set by BMC bioinformatics). [file 1471-2105-5-147-S2.bz2 › chilibotAdditionalFile2/dip10/465ID9660793E10492/html/CXCR3_CXCL10.html]

 


 **CXCR3** and **CXCL10** 
  
Found 773 abstracts in PubMed, retrieved 10.  
 

 What does Google say? 
 PDF only 
| .edu only 

---

**Interactive relationship** (e.g. stimulation, inhibition, etc)

**Neutral relationship**- The chemokine receptor  **CXCR3**  is predominantly expressed on T lymphocytes, and its agonists CXCL9,  **CXCL10**  and CXCL11 are IFN gamma inducible chemokines that promote Th1 responses.  Ref: 12884299 Eur J Immunol, 2003

**Non-interactive relationship** (e.g. studied together, co-existance, homology, etc.)

- Correspondingly, the rate or intensity of inflammation in IL 10 mice treated with anti IP 10  [ **CXCL10** ]   [ **CXCR3** ]  Abs showed improved scoring of inflammation, compared with control IL 10 mice.  Ref: 12874231 J Immunol, 2003
- This study provides important and novel information regarding IP 10  [ **CXCL10** ]   [ **CXCR3** ]  as a target for the treatment of colitis.  Ref: 12874231 J Immunol, 2003
- This work compares the proliferative, IgM, IL 6 and IP 10  [ **CXCL10** ]   [ **CXCR3** ]  response of PBMC from normal donors to a diverse panel of CpG ODNs.  Ref: 12869028 Clin Exp Immunol, 2003
- Specifically, IP 10  [ **CXCL10** ]   [ **CXCR3** ]  inhibition in IL 10 mice attenuates the associated increases in serum and or local amyloid A, IL 2, IL 6, TNF alpha, IFN gamma, IL 1alpha, and IL 1beta with colitis as compared with IL 10 mice that develop colitis similar to human Crohn s disease.  Ref: 12874231 J Immunol, 2003
- Recombinant murine interferon gamma rmIFN gamma inhibited the growth of EMT6 cells, but not K1735 cells in vitro, and strongly induced the expression of the antiangiogenic chemokine interferon inducible protein 10  [ **CXCL10** ]  IP 10  [ **CXCL10** ]   [ **CXCR3** ]  by both cell lines.  Ref: 12866028 Int J Cancer, 2003
- High levels of RANTES CCL5, MCP 1 CCL2, IP 10  [ **CXCL10** ]   [ **CXCR3** ]   **CXCL10** , and Mig CXCL9 simultaneously with mononuclear cell infiltration in the lungs was found.  Ref: 12875978 Am J Pathol, 2003
- Interferon gamma inducible protein 10  [ **CXCL10** ]  IP10  [ **CXCL10** ]   [ **CXCR3** ]  and monokine induced by interferon gamma MIG positive H& ; RS cells were detected in all 10 HL MC but only in 5 of 10 cases of HL NS and 2 of 9 cases of HL like ATLL.  Ref: 12866030 Int J CancerInt J Cancer, 2003
- In addition, IL 12 inhibits tumor angiogenesis mainly through IFNgamma dependent production of the chemokine IP10  [ **CXCL10** ]   [ **CXCR3** ] .  Ref: 12871184 Curr Pharm Des, 2003
- CCR3 functional responses are regulated by both  **CXCR3**  and its ligands CXCL9,  **CXCL10**  and CXCL11.  Ref: 12884299 Eur J Immunol, 2003
- Consistent with these results, infected cells from WT mice preferentially migrated in response to IP 10  [ **CXCL10** ]   [ **CXCR3** ]   **CXCR3**  ligand, while those from GKO mice migrated in response to eotaxin CCL11 CCR3 ligand.  Ref: 12875978 Am J Pathol, 2003
- IFN gamma inducible protein 10 IP 10  [ **CXCL10** ]   [ **CXCR3** ]  and  **CXCR3**  are highly expressed at sites of colitis.  Ref: 12874231 J Immunol, 2003
- However, 2 of 5 cases of HL like ATLL with EBV infection and 2 of 2 HL NS with EBV had IP10  [ **CXCL10** ]   [ **CXCR3** ]  MIG positive H& ; RS cells.  Ref: 12866030 Int J CancerInt J Cancer, 2003
- 2 Interferon IFN inducible anti viral proteins, including IFIT1, OAS1 and Mx1, and Th1 chemoattractant IP 10  [ **CXCL10** ]   [ **CXCR3** ] , were up regulated predominantly 6 to 24 hours after stimulation.  Ref: 12887736 BMC ImmunolBMC Immunol, 2003
- Our findings show that IP 10  [ **CXCL10** ]   [ **CXCR3** ]  significantly contributes to the development of Th1 and inflammatory responses.  Ref: 12874231 J Immunol, 2003
